# Clinical efficacy and feasibility of laser correction technology with an ordinary laser pen and surgical instrument box in open-wedge high tibial osteotomy

**DOI:** 10.1186/s12891-022-05993-4

**Published:** 2022-11-26

**Authors:** Zhe Xu, Guang Tian, Ruguo Zhang, Zhanyu Wu, Chen Liu, Chuan Ye

**Affiliations:** 1 Department of Orthopedics, Guihang Guiyang 300 Hospital, Guiyang, 550004 China; 2 National-Local Joint Engineering Laboratory of Cell Engineering and Biomedicine, Guiyang, 550004 China; 3 Department of Orthopedics, Affiliated Hospital of Guizhou Medical University, Guiyang, 550004 China; 4 China Orthopedic Regenerative Medicine Group (CORMed), Hangzhou, 310000 China

**Keywords:** Laser navigation, Leg alignment, Open-wedge high-tibial osteotomy (OWHTO), Femoral tibial angle

## Abstract

**Background:**

The clinical outcomes of open-wedge high tibial osteotomy (OWHTO) for medial knee osteoarthritis primarily depend on the corrective precision. The present study aimed to determine the efficacy and feasibility of laser correction technology with an ordinary laser pen and surgical instrument box.

**Methods:**

This prospective and randomized trial included 71 patients randomly divided into laser (*n* = 36) and traditional groups (*n* = 35). In the laser group, the hip centre, knee (Fujisawa point), and ankle centre were located preoperatively using the surgical instrument box lid. The leg was aligned with an ordinary laser pen. In the traditional group, the lower limb alignment was corrected by a metal cable. Radiation exposure, operative time, and rate of outliers (lower limb force line does not pass through 62–66% of the lateral tibial plateau) were evaluated. The visual analogue scale (VAS) and Western Ontario and McMaster Universities Osteoarthritis Index (WOMAC) scores were recorded. After 24 months, the femoral tibial angle (FTA), medial proximal tibial angle (MPTA), and posterior slope angle (PSA), were recorded. The Kaplan-Meier method was used to evaluate the survival time of affected knees, and postoperative complications were recorded.

**Results:**

The radiation exposure, operative time and rate of outliers were lower in the laser correction group (*P* < 0.05). Six months postoperatively, the VAS and WOMAC scores were significantly improved in both groups (*P* < 0.001). At 24 months, the FTA, MPTA, and PSA were corrected in both groups (*P* < 0.001). There were no differences in the postoperative knee survival time from OWHTO to knee arthroplasty between the groups or postoperative complications (*P* = 0.53; *P* = 0.61).

**Conclusions:**

Laser correction technology can effectively reduce radiation exposure, the operative time, and the rate of outliers (trial identification number (retrospectively registered): ChiCTR2200060480; date of register: 03/06/2022).

## Background

Open-wedge high tibial osteotomy (OWHTO) is a widely performed procedure for the treatment of medial knee osteoarthritis [1–3]. It is best indicated for young and active people with moderate arthritis (patients with a narrow joint space and no obvious osteophytes or joint instability). The goal of the surgery is to correct the lower limb mechanical axis by osteotomy of the proximal tibia to reduce the abnormal stress distribution in the knee joint and transfer the compressive loads from the medial compartment to the relatively normal lateral compartment. At present, the desirable correction of lower limb alignment is controversial; some recent studies have shown that the postoperative mechanical axis should be shifted to 50–55% of the tibial plateau width [4], but most articles have recommended a range of correction of 62–66% of the lateral knee platform [5]. The precision of correction is more stringent than that of total knee arthroplasty (TKA), and the error is generally required to be controlled within ±1° [6]. Studies have suggested that the medium-term knee survival time of OWHTO can reach 5–10 years [7]. Over- and undercorrection can affect long-term clinical outcomes [8, 9].

At present, intraoperative assessments of correction precision primarily depend on X-ray and repeated measurements of the metal pole or cable [10, 11]. Metal poles or cables are used as surface references of the hip and ankle centres. By opening the osteotomy zones, leg alignment can be corrected; however, repeated fluoroscopy results in a prolonged operation time, increased radiation exposure and fatigue among surgeons and imaging technicians. Moreover, the precision of these measurements is limited, and there is a high incidence of over- and undercorrection. Therefore, overcorrection and undercorrection often occur after OWHTO [12–14]. In recent years, computer navigation technology has improved the precision of correction [10, 15–17], but due to the complexity of the technical operation, the long learning curve and the high cost of the equipment, the implementation of OWHTO in primary hospitals is limited [18, 19].

Inspired by common laser pens and surgical instrument boxes with grids, this study utilized a laser beam in a laser correction system because it is monochromic, nondivergent, and advanced in a straight line [20, 21]. In addition, with the help of a grid-shaped instrument box, the hip centre, knee (Fujisawa point), ankle centre and proximal tibial osteotomy channel can be located efficiently and accurately.

Only a few studies have used laser correction technology to position the force lines in hip and knee arthroplasty and orthopaedic percutaneous surgeries [22–24]. However, this technology has been widely used in urology, oral surgery and ophthalmology applications and has demonstrated satisfactory clinical effects [25–27]. This is the first time that laser correction technology has been applied in OWHTO. The purpose of the study, therefore, was to investigate the feasibility and efficacy of laser correction technology using an ordinary laser pen and surgical instrument box in the operating room. The hypothesis of the present study was that laser correction technology can more accurately correct the mechanical force line of the lower limb, improve operation efficiency, reduce radiation exposure, and improve the rate of outliers.

## Materials and methods

All experimental protocols were approved by the Ethics Committee of the Affiliated Hospital of Guizhou Medical University, and informed consent was obtained from all patients. All methods were carried out in accordance with relevant guidelines and the Helsinki Declaration. The patients included 22 males and 49 females aged 50–60 years; 39 operations were performed on the left knee, and 32 were performed on the right knee (Table 1).

**Table 1:** Demographics

General case	Traditional group	Laser group	***P value***
Sex (male/female)	10/25	12/24	0.80
Age (years)	54.51 ± 0.31	55.52 ± 0.21	0.53
K/L grade (II/III)	17/18	15/21	0.64
Affected side (left/right)	19/16	20/16	1.00
**Radiological angle**			
MPTA (°)	81.03 ± 2.36	81.25 ± 1.61	0.65
FTA (°)	181.17 ± 3.15	180.31 ± 2.72	0.22
PSA (°)	8.46 ± 0.62	8.47 ± 0.39	0.98

No significant differences in age, sex, or Kellgren-Lawrence (*K/L*) grade, medial proximal tibial angle (*MPTA*), femoral tibial angle (*FTA*), or posterior slope angle (*PSA*) were observed between the two groups (*P* > 0.05)

**Fig. 1:**
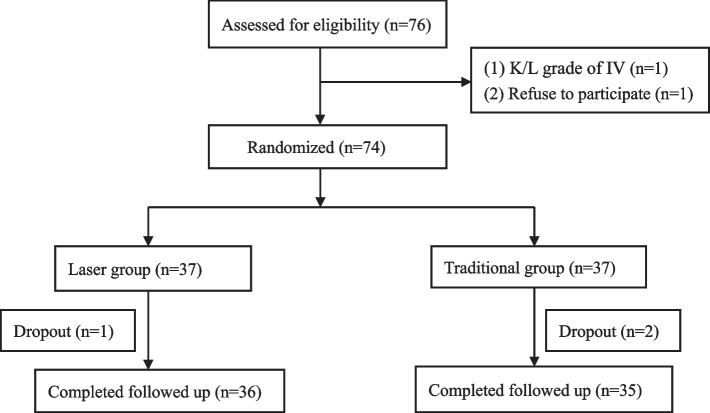
Flow chart showing the randomization process

The inclusion criteria were as follows (Fig. 1): (1) age younger than 60 years; (2) basically normal mobility of the knee joint; (3) flexion contracture deformity < 10°; (4) 5° < varus deformity < 20°; (5) proximal tibial angle < 85°; and (6) normal lateral meniscus and cartilage function. The exclusion criteria were as follows: (1) Kellgren-Lawrence (K/L) grade IV; (2) secondary osteoarthritis (infectious arthritis, rheumatoid arthritis, haemophilic arthritis, etc.); (3) meniscus and ligament injury; and (4) severe cardiovascular disease, liver or kidney disease, endocrine system disease, blood system disease or mental disease.

### Surgical procedure and rehabilitation

**Fig. 2:**
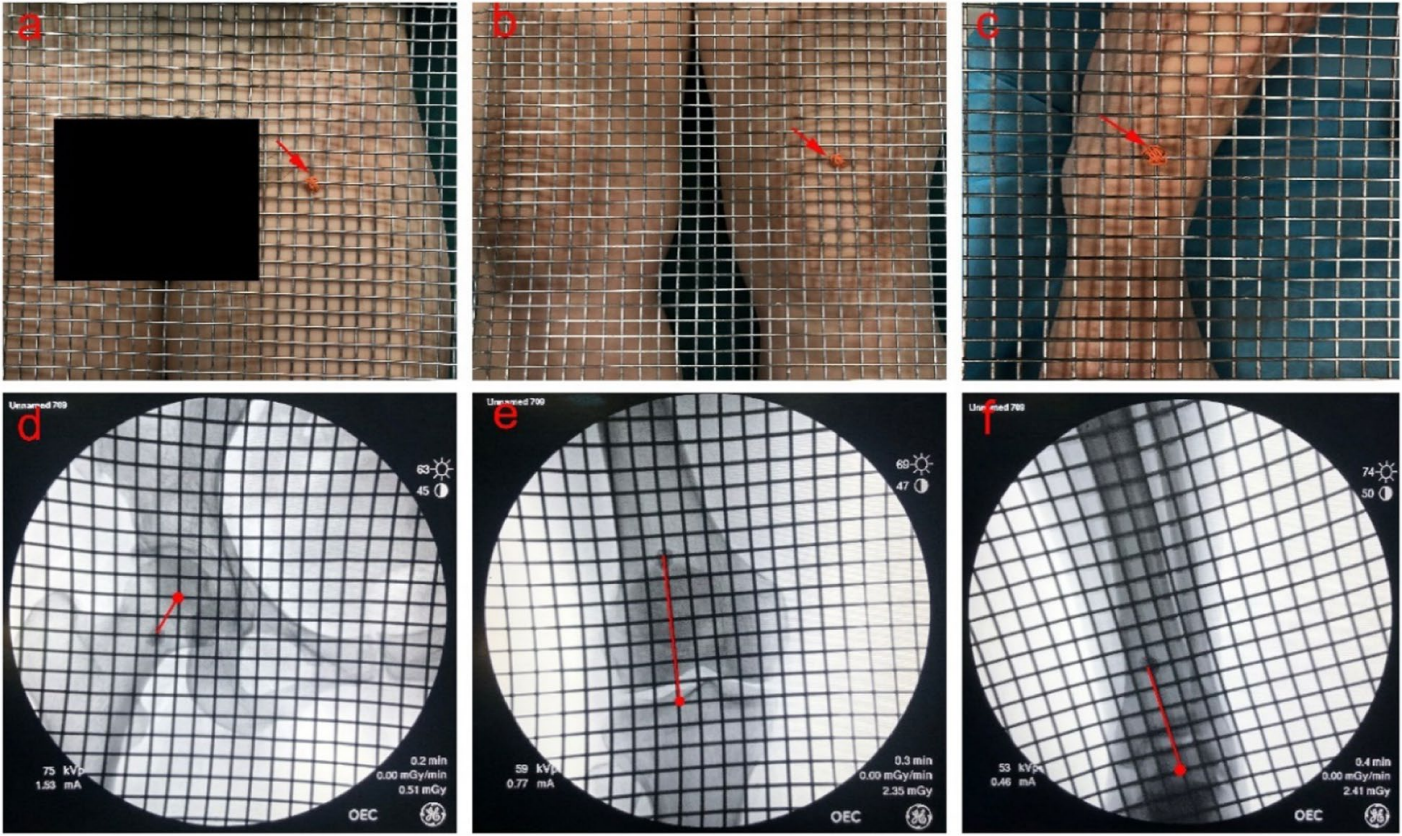
**a**~**c** An orange coil (arrow) was used as a reference point. **d**~**f** The hip centre, knee (Fujisawa point), and ankle centre (red points) determined using X-ray fluoroscopy. The red line represents the distance from the reference point (orange coil) to the target points. This method can save time and allow the projection of the hip centre, Fujisawa point, and ankle centre to be located more easily

All operations were completed by experienced senior doctors, and each operation was carried out in strict accordance with the surgical indications and specifications to reduce the over- and undercorrection caused by surgical indications or surgical error. The surgical instrument box needed to be properly placed horizontally, the orange coil was used as a reference point to locate the body surface projection of the hip centre, knee (Fujisawa point) and ankle centre (Fig. 2a~c), and a protuberant device was placed at the hip joint centre so that it could be identified during the operation. A surgical instrument box (the edge distance of the square was 0.5 cm) and X-ray fluoroscopy (Fig. 2d~f) were used to locate the hip centre, knee (Fujisawa point), and ankle centre.

**Fig. 3:**
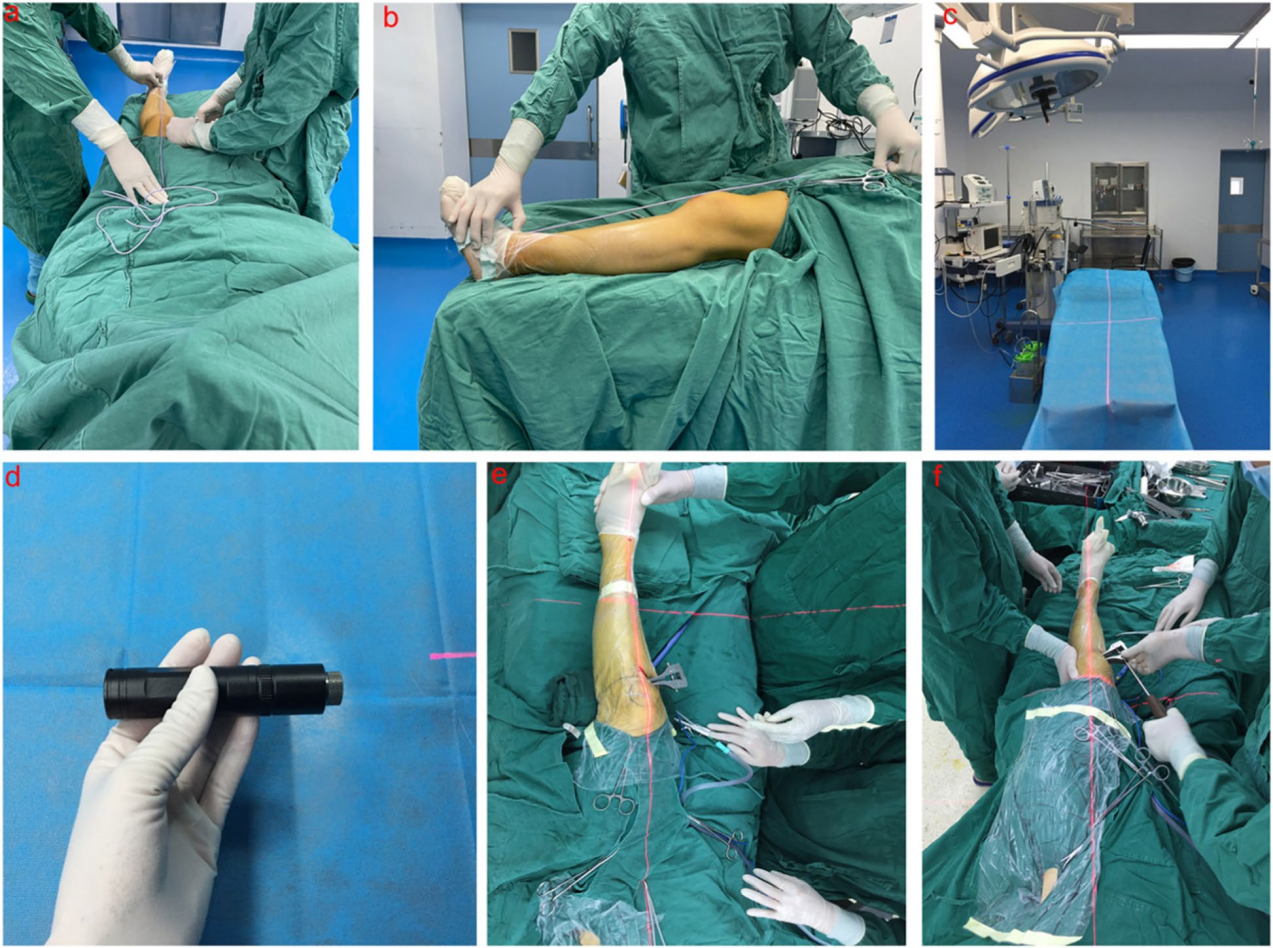
**a**, **b** The lower limb mechanical axis was corrected using the metal cable. **c**, **d** Common laser pen and installation location in the operation room. **e**, **f** Correction of the lower limb mechanical axis using the laser beam from a common laser pen

A thigh tourniquet was applied with the patient in the supine position. An approximately 4- to 5-cm incision was made longitudinally at the 3- to 4-cm medial portion of the anterior ridge of the tibia. The medial collateral ligament was released from the tibia, and the semitendinosus and gracilis tendons were preserved. A single-plane osteotomy was performed using an osteotome and a bone saw, leaving the lateral cortex intact to serve as a hinge. The osteotomy channel is located at the distal end of the tibial tubercle to minimize the impact on the patella position [28]. After the osteotomy channel was established, the distractor was used to correct the leg alignment. During the operation, to obtain an accurate correction in the alignment of the lower limb, the patella was kept in a neutral position when correcting the alignment of the lower limb. In the traditional group, a metal cable was mainly used as the reference tool to correct the alignment of the lower limb (Fig. 3a, b). The procedure was as follows: Find the centre of the ankle joint and hip joint through the C-arm machine, make the metal cable pass through the centre of the ankle and hip joint, and make the metal cable pass through the knee (Fujisawa point) of the knee joint through the C-arm fluoroscopy and the distractor. During operations, iatrogenic proximal tibial fracture should be avoided with the help of a C-arm machine. In the laser group, a laser pen (DL552001, Deli, Jiangsu, China) was used as a tool to correct the alignment of the lower limb; the laser pen was installed at the centre of the surgical operation light using a clip (Fig. 3c). Meanwhile, the laser pen was tested to determine whether it functioned well (Fig. 3d). Before correction, the laser beam should pass through the projection of the hip and ankle (Fig. 3e). After correction, if the laser beam from the laser pen passed through the projection of the hip centre, knee (Fujisawa point), and ankle centre, the mechanical axis was considered to be corrected (Fig. 3f). When establishing the proximal tibial osteotomy channel with a Kirschner wire and correcting the lower limb force line with a laser pen, the C-arm machine was used to observe the osteotomy position and whether there was iatrogenic proximal tibial fracture. The laser beam source had a wavelength of 650 ± 10 nm. The maximum output was 5 mW, and the laser grade was 3R. With the exception of the possibility of retinal damage when an individual stares directly at the beam, laser beams are harmless to humans. The prepared autogenous iliac bone block was inserted into the osteotomy channel, and the osteotomy was supported using a locking plate designed for OWHTO (TomoFix, Synthes, Bettlach, Switzerland). Postoperatively, partial weight-bearing was limited for 6 weeks after surgery, and full weight-bearing was permitted from 6 weeks after the operation. Active knee motion exercises were started postoperatively after the removal of the drainage tube.

### Outcomes

Neither the patients nor the researchers knew the group assignments for the trial. All data were evaluated by independent physicians who remained blinded to the study. Through a simple random remainder grouping method, the patients who met all the inclusion criteria were randomly assigned to the traditional and laser groups. The visual analogue scale (VAS) and Western Ontario and McMaster Universities Osteoarthritis Index (WOMAC) were recorded for both groups before and at 24 months after the operation. The scores were mainly determined by telephone questionnaires. Radiographs, including full-length X-ray images of the lower limb and anteroposterior and lateral images of the knee, were evaluated at 1, 6, 12, and 24 months postoperatively and on a routine basis thereafter. The medial proximal tibial angle (MPTA), femoral tibial angle (FTA), and posterior slope angle (PSA) were measured preoperatively and postoperatively using a picture archiving and communication system (Hechuang Digital Technology Company, Beijing, China) and anteroposterior and lateral X-ray images of the knee to evaluate the efficiency of correction. MPTA is the medial angle formed between the tangent of the medial and lateral tibial plateau and the tibial anatomical axis. The FTA is the lateral angle formed by the intersection of the femoral anatomical axis and the tibial anatomical axis at the centre of the knee joint. On standard lateral radiographs of the knee, PAS is the angle formed by the tangent of the medial and lateral tibial platforms and the perpendicular of the tibial anatomical axis. The radiation dose (mSv), operative time (minutes) and postoperative complications were recorded. At 1 and 24 months postoperatively, the rate of outliers, defined as the lower limb force line not passing through 62–66% of the lateral tibial plateau, was evaluated. The knee survival time from OWHTO to knee arthroplasty was recorded.

### Power calculation and statistical analysis

The sample size estimation was calculated using PASS 15.0 software. A minimum sample size of 56 patients was required (or 28 patients per group) based on a study power of 90% (*β* = 0.10), a false-positive rate of 5% (*α* = 0.05) and effect size (Cohen’s *d* = 0.89) in the traditional and laser groups at 24 months postoperatively for VAS scores, according to a pilot study. Predicting a 20% dropout rate, we enrolled approximately 36 patients per group at baseline.

The data were analysed by SPSS 25.0 (IBM company, Chicago, USA). All data were normally distributed. All measurement data are expressed as the means ± standard deviations. Repeated-measures analysis of variance was used for VAS and WOMAC scores between time points within the same group. The least significant difference (LSD) or Tamhani test was used to compare groups. Paired or independent-samples t tests were used for comparisons between the two groups. The Kaplan-Meier method was used to evaluate the survival time of the affected knees. No significance level was *P* > 0.05.

## Results

### Clinical scores

#### Pain scores

**Fig. 4:**
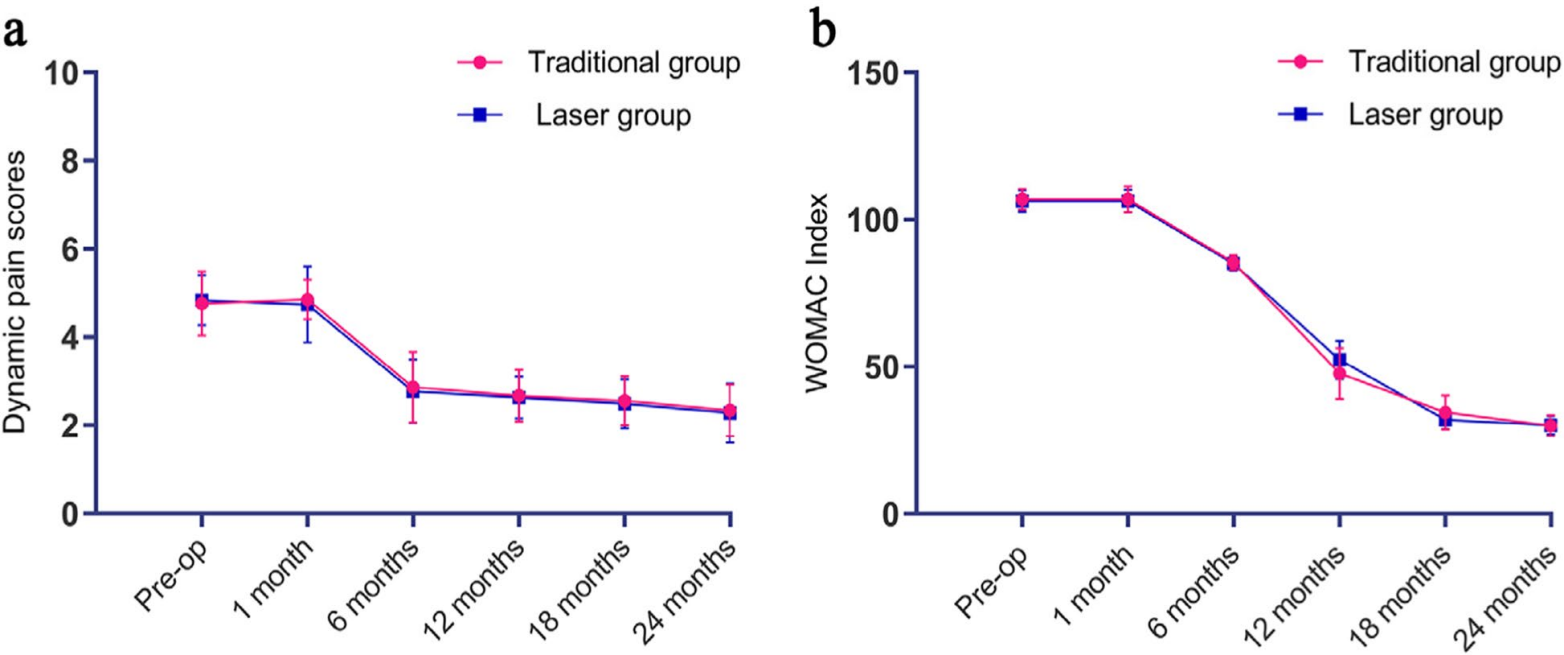
**a**, **b** The pain level and knee function in both groups showed significant improvements at 6, 12, 18 and 24 months (*P* < 0.001), but no significant differences were observed between the two groups (*P* > 0.05)

One month after the operation, the average pain score (Fig. 4a) in the traditional group changed from 4.76 [95% CI 4.66–4.86] to 4.85 [95% CI 4.74–4.95] (*P* = 0.960) and that in the laser group changed from 4.84 [95% CI 4.74–4.94] to 4.74 [95% CI 4.64–4.85] (*P* = 0.925). The VAS scores in the two groups showed significant improvements at 6, 12, 18, and 24 months after the operation (*P* < 0.001), but no significant differences were found between the groups (*P* > 0.05).

#### Functional scores

One month after the operation, the average WOMAC index (Fig. 4b) in the traditional group changed from 106.98 [95% CI 106.86–107.09] to 106.95 [95% CI 106.81–107.08] (*P* = 1.000); in the laser group, the index changed from 106.26 [95% CI 106.15–106.38] to 106.30 [95% CI 106.17–106.43] at 1 month postoperatively (*P* = 1.000). At 6, 12, 18 and 24 months after the operation, the WOMAC index showed significant improvements (*P* < 0.001), but there were no significant differences between the two groups (*P* > 0.05).

### Postoperative complications

Postoperative complications were observed from preoperatively to 12 months postoperatively. Complications mainly originated from delayed healing of the incision. The operative time was longer in the traditional group, which may be the main reason for the increased incidence of delayed incision healing in the traditional group (Table 2). There was no difference in postoperative complications between the two groups (*P* = 0.61).

**Table 2:** Postoperative complications

Postoperative complications	Traditional group	Laser group	***P value***
Infection	0	0	
Incision exudation	0	0	
Delayed healing of incision	2	1	
Fracture of the lateral cortex or of the proximal fragment of the tibia	0	0	
Nonunion of bone	0	0	
Lower limb ischaemia	0	0	
Sensory disturbance	0	0	
Deep vein thrombosis	0	0	
Incidence of complications	2/35	1/36	*P* = 0.61

There was no difference in postoperative complications between the traditional and laser groups (*P* = 0.61)

### Radiological outcomes

#### MPTA, FTA, and PSA

The MPTA, PSA, and FTA (Table 3) were significantly corrected in both groups at 24 months (*P* < 0.001), and no significant differences in the MPTA, FTA or PSA were observed between the groups (*P* > 0.05).

**Table 3:** Anatomic angle

	Traditional group	***P value***	Laser group	***P value***
Anatomic angle	Preoperatively	24 months	Preoperatively	24 months
MPTA (°)	81.03 ± 2.36	91.14 ± 3.05 0.00	81.25 ± 1.61	90.89 ± 2.98 0.00
FTA (°)	181.17 ± 3.15	172.63 ± 2.57 0.00	180.31 ± 2.72	172.67 ± 2.76 0.00
PSA (°)	8.46 ± 0.62	10.07 ± 0.67 0.00	8.47 ± 0.39	10.58 ± 0.78 0.00

The medial proximal tibial angle (*MPTA*), femoral tibial angle (*FTA*), and posterior slope angle (*PSA*) were significantly corrected after 24 months in both groups (*P* = 0.00)

#### The rate of outliers

**Fig. 5:**
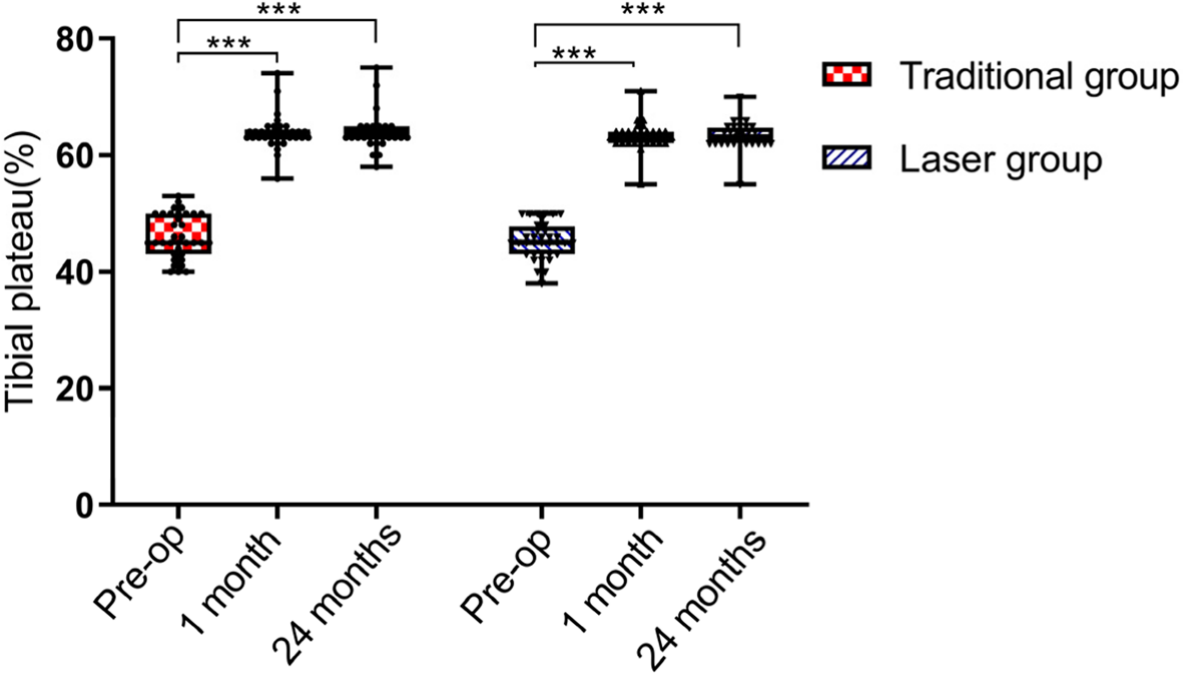
At 1 and 24 months after the operation, varus deformities were significantly corrected in both groups (*P* = 0.000), but the correction precision did not significantly differ between the groups (*P* = 0.16; *P* = 0.35, respectively). One month after the operation, there were 2 cases of overcorrection and 2 cases of undercorrection in the traditional group and 1 case of overcorrection and 1 case of undercorrection in the laser navigation group. The correction efficacy did not change at 24 months postoperatively. After 24 months, the rate of outliers in the traditional and laser groups was 11.43 and 5.56%, respectively (*P* = 0.00)

At 1 and 24 months postoperatively, varus malalignment in the traditional and laser groups was significantly corrected (*P* = 0.000). The mechanical axis shifted to cross the lateral aspect of the tibial plateau at a point located at 64.0 0 ± 3.12% and 63.00 ± 2.75% 1 month postoperatively and at 64.14 ± 3.28% and 63.64 ± 1.80% at 24 months postoperatively in the two groups (Fig. 5). At 1 and 24 months after the operation, no significant difference was found between the two groups in the accuracy of leg alignment correction (*P* = 0.16; *P* = 0.35), but the rate of outliers in the traditional and laser groups was 11.43 and 5.56%, respectively, at 24 months postoperatively (*P* = 0.00).

#### Perioperative situation

**Fig. 6:**
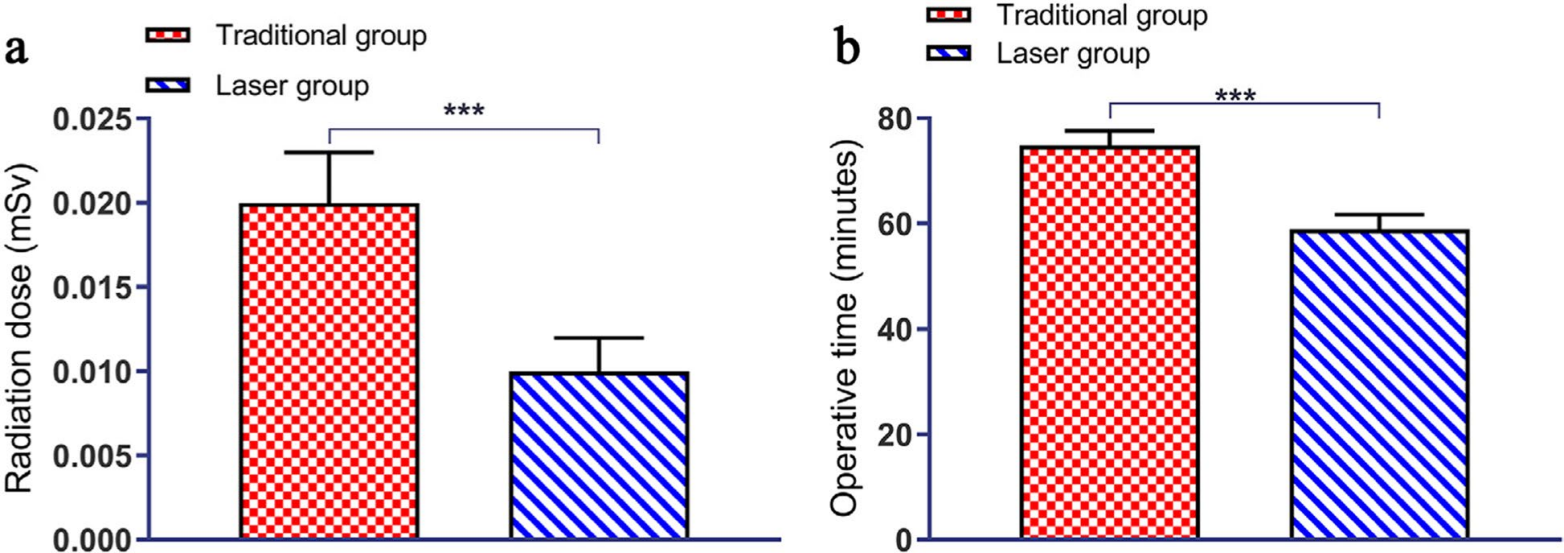
**a**~**b** The radiation dose and operative time were lower in the laser group than in the traditional treatment group (*P* < 0.05)

The radiation dose (Fig. 6a) was 0.19 [95% CI 0.18–0.21] mSv and 0.11 [95% CI 0.10–0.11] mSv in the traditional and laser groups, respectively (*P* = 0.00); the operative time (Fig. 6b) was 74.89 [95% CI 73.95–75.82] min and 58.92 [95% CI 57.30–60.54] min, respectively (*P* = 0.00).

#### Survival analysis

**Fig. 7:**
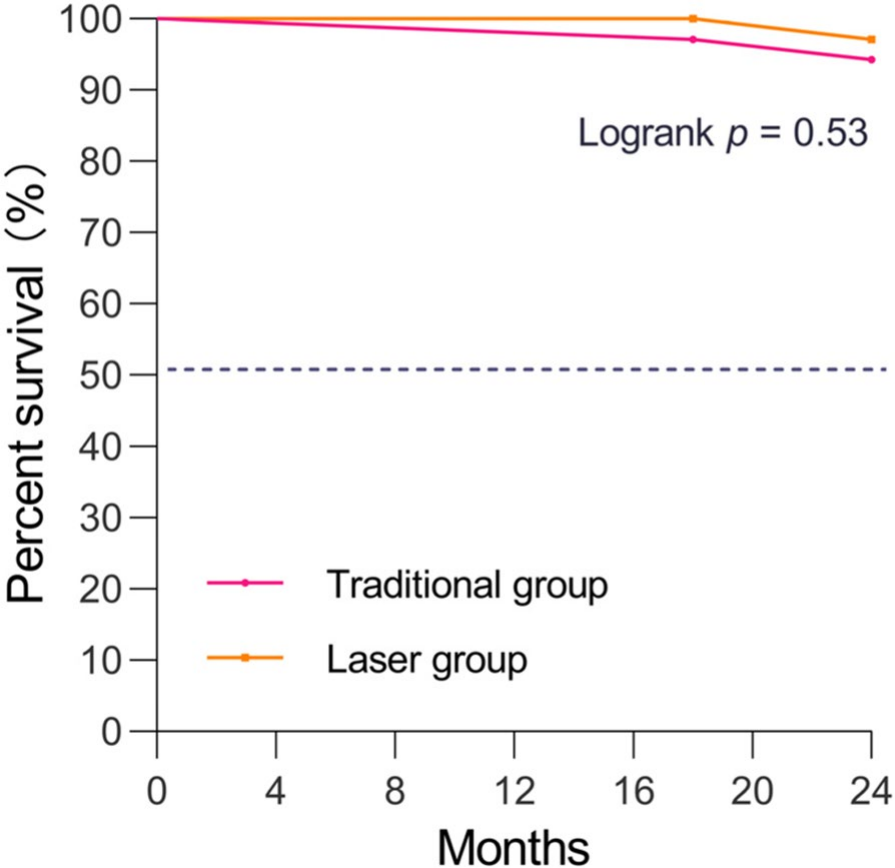
There was no difference in the postoperative knee survival time between the traditional and laser groups (*P* = 0.53)

The survival time from OWHTO to knee arthroplasty of the affected knee 24 months after the operation was analysed by the Kaplan–Meier method. Survival analysis (Fig. 7) showed that 2 patients in the traditional group underwent knee replacement at 18 and 24 months postoperatively, and 1 patient in the laser navigation group underwent knee arthroplasty at 18 months postoperatively. In the traditional group, the reason for knee replacement was severe tibial plateau compression fracture in 1 patient and no obvious improvement in knee pain in 1 patient. In the laser navigation group, the meniscus and cartilage of the affected knee sustained severe trauma in a traffic accident, and the resulting pain was not relieved by arthroscopic treatment. After consideration, we performed knee arthroplasty in these patients. There was no difference in the survival time of the knee between the two groups (*P* = 0.53).

## Discussion

The most important finding of this prospective randomized controlled study was that laser correction technology can effectively reduce radiation exposure, the operative time, and the rate of outliers. Laser correction technology is therefore advantageous compared with traditional positioning technology.

In our study, knee pain and function, as evaluated by the VAS and WOMAC scores, continued to improve for 12 months after the operation. Because the time of bone remodelling with the TomoFix plate is appropriately 6–18 months, according to the needs of patients, we chose to remove the implants at 18 months postoperatively. Therefore, the improvement in knee function from 12 to 24 months postoperatively can be explained by implant removal [1].

Traditional technology involves the use of a metal cable to correct the lower limb mechanical axis, which inevitably negatively affects the operation. The metal cable can be deformed by external forces, which ultimately affects the accuracy of the lower limb alignment correction [29]. Although computer navigation technology has basically solved the problem of correction accuracy [29–33], the equipment is expensive; in addition, this technology increases the complexity of the operation and thus is not suitable for implementation in areas with limited medical resources. Laser correction technology combined with a mesh surgical instrument box can effectively improve the accuracy and stability of surgical correction. Laser correction technology is easy to operate, and beginners can quickly master its use. Moreover, similar to other computer navigation technologies, it can effectively reduce the probability of outliers since the laser has a strong anti-interference ability and will not interfere with the positioning accuracy of the operator [20]. Moreover, an intuitive lower limb mechanical axis can be created when the operator uses the laser beam. The application and maintenance costs are very low, and the operation is simple and convenient to perform. It is suitable for use in hospitals at any level [18, 19].

Radiation exposure contributes to iatrogenic injury. Iatrogenic radiation injury is becoming a problem of increasing concern in the international medical community [34–39]. The application of metal cables in traditional medial high tibial osteotomy is complicated and requires medical personnel to carry out repeated X-ray fluoroscopy examinations to verify the precision of the mechanical axis correction, which inevitably exposes both medical workers and patients to radiation [17, 34, 36]. Laser correction technology solves the problem of insufficient deformation resistance of traditional cables. The laser cannot deform due to interference from external forces. Because the laser beam emitted by the laser pen is always in a straight line, laser correction technology has the characteristics of accuracy and anti-interference [20, 21]. It has higher precision and stability than traditional metal cables and can effectively reduce the incidence of undercorrection and overcorrection.

Previous comparisons of computer navigation technology with traditional treatment in terms of the operative time and radiation exposure have indicated that the former is advantageous [17, 33]. The findings revealed that the operation time of the laser correction group was significantly shorter than that in the traditional group, primarily because the time required for positioning the surface references for the lower limb force line and proximal tibial plateau osteotomy decreased when the surgical grid-shaped instrument box was used. Since laser correction will not affect the operation and its application is simple and convenient, it can reduce the operative time.

More X-ray fluoroscopy examinations were required in the traditional group, as well as repeated adjustments of the mechanical axis during the operation, which increased the radiation exposure and operative time. Although some animal experiments have shown that radiation exposure can have a negative impact on incision healing, the radiation dose in this study was so low that it could not delay incision healing [40, 41]. According to a previous study [42], a longer operation may contribute to an increased incidence of delayed incision healing, as was observed in the traditional group. However, there was no significant difference in postoperative complications between the two groups due to delayed incision healing.

This investigation suggests that the main reason for knee replacement was unexpected trauma. While the effect in one patient in the traditional group was unsatisfactory, the survival time of the affected knee after OWHTO was satisfactory in most patients, which is consistent with most clinical research results [7, 43].

However, this study has some limitations. First, the sample size was small, and more cases need to be studied to increase the quality of the evidence. Second, the status of the knee cartilage was not observed postoperatively, which requires attention in the next step of the research. Finally and most importantly, the observation period was short, and a longer follow-up period is needed to determine the clinical efficacy of simple technology. A follow-up period of 2 years does not allow us to draw conclusions about the long-term clinical outcomes of our patients, including the need to undergo total knee replacement in the future.

## Conclusions

Laser correction technology can effectively reduce radiation exposure, operative time, and the rate of outliers.

## Data Availability

The datasets of the current study are available from the corresponding author upon reasonable request.

## Abbreviations

FTA: Femoral tibial angle
PSA: Posterior slope angle
MPTA: Medial proximal tibial angle
OWHTO: Open-wedge high-tibial osteotomy
